# P-2117. Infections Caused by Filamentous Basidiomycetes, an Emerging Fungal Pathogens : A Retrospective Study from King Chulalongkorn Memorial Hospital, Thailand

**DOI:** 10.1093/ofid/ofaf695.2281

**Published:** 2026-01-11

**Authors:** Chaianant Leelabooranasak, Arsa Thammahong, Jakapat Vanichanan

**Affiliations:** King Chulalongkorn Memorial Hospital - Bangkok (Thailand), Thonburi, Krung Thep, Thailand; King Chulalongkorn Memorial Hospital - Bangkok (Thailand), Thonburi, Krung Thep, Thailand; Chulalongkorn University, Bangkok, Krung Thep, Thailand

## Abstract

**Background:**

Filamentous Basidiomycetes fungi are abundant in environment and rarely cause invasive infections in human. Currently, those fungi are increasingly recognized as emerging opportunistic pathogens, particularly in immunocompromised individuals. Clinical data on these infections remain limited.

**Figure:**
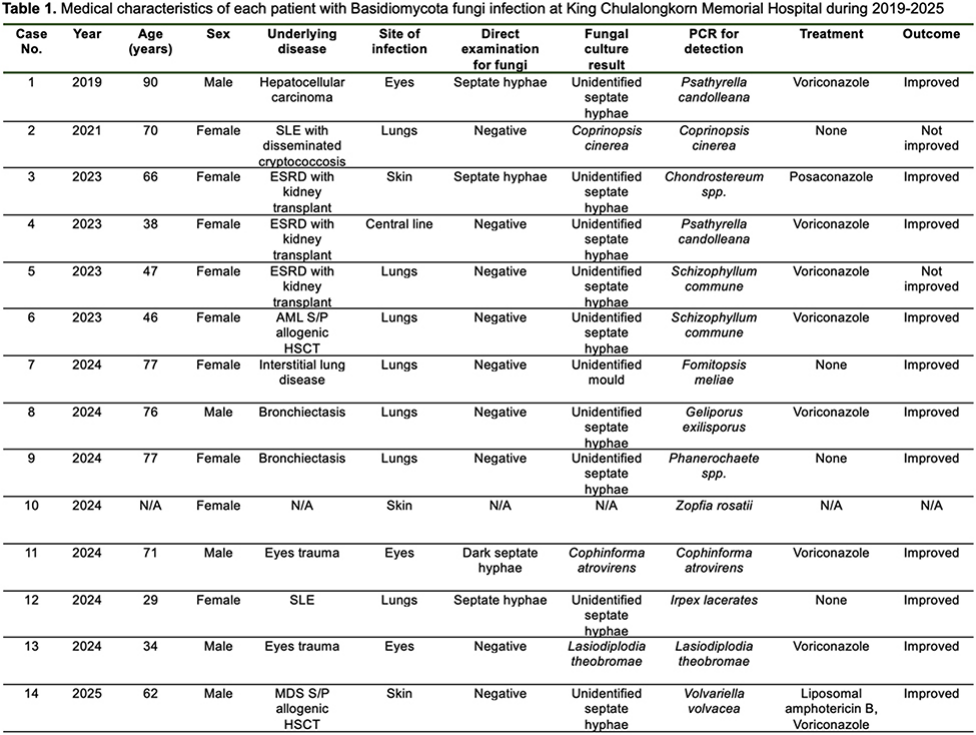
SLE = systemic lupus erythematosus, ESRD = end-stage renal disease, AML = acute myeloblastic leukemia, HSCT = hematopoietic stem cell transplantation, MDS = myelodysplastic syndrome

**Methods:**

A retrospective analysis was conducted in King Chulalongkorn Memorial Hospital, a tertiary-care hospital in Bangkok Thailand. We included patients who had isolated from clinical specimen which subsequently identified as filamentous Basidiomycetes fungi between 2019 – 2025. Species identification was performed using PCR targeting the ITS region with Sanger sequencing. Clinical characteristics and treatment outcomes were collected.

**Results:**

A total of 14 isolates of filamentous Basidiomycetes fungi were identified from clinical specimens including bronchoalveolar lavage (BAL; n = 6), ocular specimens (n = 3), tissue/pus (n = 3), endotracheal aspirate (n = 1) and central line (n = 1). Among them, 4 isolates were considered as contamination and was not treated. The most common site of infection was lungs (n = 3) and ocular (n = 3), followed by skin (n = 2) and central line (n = 1). Causative organisms were demonstrated in table 1. Underlying conditions were kidney transplantation (n = 3), hematopoietic stem cell transplant (n = 2), trauma (n = 2), structural lung disease (n = 1) and malignancy (n = 1). Majority of patients (77%) received voriconazole as antifungal therapy while posaconazole and combination amphotericin B + voriconazole were prescribed in 1 patient (23%) each. Overall, clinical improvement was achieved in 11 patients (84.6%), while 2 patients did not respond.

**Conclusion:**

Our study highlights the emerging clinical relevance and diversity of Basidiomycota fungi. Molecular identification is important method to identify pathogen. Without clear treatment recommendation, voriconazole and posaconazole appeared to be an effective treatment.

**Disclosures:**

All Authors: No reported disclosures

